# Herpesvirus-Associated Acute Urticaria: An Age Matched Case-Control Study

**DOI:** 10.1371/journal.pone.0085378

**Published:** 2013-12-27

**Authors:** Arianna Mareri, Stuart P. Adler, Giovanni Nigro

**Affiliations:** 1 Pediatric Unit and School, University of L'Aquila, San Salvatore Hospital, L’Aquila, Italy; 2 Department of Microbiology, Virginia Commonwealth University, Medical College of Virginia Campus, Richmond, Virginia, United States of America; University of Liverpool, United Kingdom

## Abstract

**Background:**

Acute and recurrent acute urticaria are often associated with multiple factors including infections and recent data suggest a role for herpesviruses.

**Objective:**

To test the null hypothesis, that is, there is no association of herpesvirus infections with urticaria.

**Methods:**

Thirty-seven patients between one month and 15 years of age were age matched to 37 controls who were healthy or had mild acute respiratory infections but without urticaria. Patients and controls were followed for 1 to 6 years. Diagnostic studies included DNA detection by real-time PCR for herpes simplex virus (HSV) types 1 and 2, Epstein-Barr virus (EBV), cytomegalovirus (CMV) and human herpesvirus-6 (HHV-6). Tests for other infections included adenovirus, parvovirus B 19, respiratory syncytial virus, influenza A, Group A streptococci, rotavirus, and parasites.

**Results:**

Specific infections were diagnosed in 26 of 37 cases and among 9 of 37 control children (P=0.0002). Single or concomitant herpesvirus infections occurred in 24 cases and in 4 controls (65% vs 11 %, p=0.0003). Cases had 10 HHV-6 infections, 8 CMV infections, 5 EBV infections, and 4 HSV-1 infections.

**Conclusion:**

Herpesvirus infections are associated with acute or recurrent acute urticaria.

## Introduction

Urticaria is a common skin reaction characterized by itchy short-lived elevated erythematous wheals anywhere on the skin. Acute urticaria is associated with symptoms for less than 6 weeks, and is considered to be recurrent acute if the duration of symptom-free periods between urticaria episodes is longer than 6 weeks [1]. Although a specific cause is not frequently identified, the onset of acute urticaria has been associated with allergic reactions to foods and drugs, contact with chemicals, physical stimuli, or infections.

Nearly all of the numerous studies reporting evidence for infectious agents triggering acute or recurrent acute urticaria are retrospective observational studies without appropriate controls or are case reports. Different studies reported a rate ranging from 37% to 58% of multiple infections among 54 or 79 patients with acute urticaria, respectively [2,3]. Another study highlighted the role of *Mycoplasma pneumoniae* infection, which occurred in 32% of 65 children with acute urticaria [4]. Other reports focused on an association between acute urticaria and streptococcal infection, hepatitis A and B viruses, parvovirus B19, cytomegalovirus (CMV), coxsackie A9 virus, enterovirus, influenza A and parainfluenza viruses [5-10]. Acute urticaria has also been observed after influenza vaccination [11]. Upper respiratory or digestive symptoms are common with acute urticaria associated with infections [10,12,13]. These studies do not provide a compelling case for causal link between a specific infection and the development of urticaria. Reports of herpesvirus infections are frequent but reports of associated urticaria are few. We thus investigated the association of herpes simplex virus (HSV) types 1 and 2, Epstein-Barr virus (EBV), CMV and human herpesvirus-6 (HHV-6) with acute or recurrent acute urticaria. 

## Methods

### Ethical statement

The study was approved by the local ethical committee named Comitato Etico del Dipartimento di Medicina Sperimentale e Sanità Pubblica, Università dell’Aquila, L’Aquila, Italy. Written consent was obtained from each subject or their parent or guardian. The local ethical committee approved the written consent process. Consent was documented in writing. 

### Subjects

A prospective study was performed from November 1, 2005 to October 30, 2010, and included 37 patients, 26 of whom were outpatients and 11 were hospitalized due to a first attack of acute urticaria. All patients had a physical examination and history that included time of onset of disease, frequency duration size and distribution of wheals, diurnal variation, shape, associated angioedema, family history of urticaria and atopy, previous or current allergies, infections, gastric/intestinal problems, use of drugs, and correlation to food. The diagnosis of urticaria was made by the appearance of circumscribed, slightly elevated, erythematous, edematous papules or wheals. Lesions varied from moment to moment and disappeared within minutes or hours. As controls 37 children who were healthy or had mild acute respiratory infections, who were predominantly siblings or playmates of the patients, were age-matched to within one year of the cases and had the same diagnostic studies. Both cases and controls were followed every 3 to 6 months for 1 to 6 years (mean: 4.2 years and 3.8 respectively).

### Laboratory investigations

Laboratory tests at enrollment for cases included complete blood counts, platelets, erythrocyte sedimentation rate (ESR), C-reactive protein (CRP), aspartate transaminase (AST), alanine transaminase (ALT), and urine examination. In all cases serum immunoglobulin (IgG, IgA, IgM, IgE) were examined. 

In both cases and controls at enrollment EBV-DNA, CMV-DNA and HHV-6-DNA were detected in the serum and saliva with the use of Real-Time polymerase-chain-reaction. IgG and IgM antibodies against HSV, EBV, CMV, and HHV-6 were examined in serum samples, using commercial enzyme-linked immunosorbent assay from Radim (Pomezia, Italy) and Sorin (Saluggia, Italy). A viral infection was considered as active if high levels of virus-specific IgM antibodies were initially detected with an increase in concentration of viral specific IgG antibodies or HSV-1/2, EBV, CMV or HHV-6 DNA was detected in at least one sample. Other enrollment studies for cases and controls included IgG and IgM anti-adenovirus, respiratory syncythial virus, influenza virus and parvovirus B19 as well as rotavirus detection in the stools. Group A streptococcal infection was diagnosed if a throat swab was positive and/or a significant ASO increase (≥ double of previous one) occurred. Specific IgG and IgM antibodies towards *Mycoplasma* and *Chlamydia pneumoniae* were performed in children with upper respiratory infections. *Helicobacter pylori* and specific IgG antibodies were tested for in children with recurrent abdominal pain. 

Allergic studies included skin prick and total and specific IgE. Skin test was determined by using undiluted commercial allergens (cow’s milk, whole eggs, chocolate, tomato, grasses, olive, Parietaria, Artemisia, Alternaria, Ambrosia, Betulla, *Dermatophagoides pteronyssinus*, *Dermatophagoides farinae*, Penicillium, dog, cat). A positive histamine control skin test was performed. A positive skin test was defined as inducing a reaction with a wheal size measuring 3 mm or more after subtraction of the control value. Specific IgE antibodies to bovine milk proteins (α-lactalbumin, β-lactoglobulin, and casein) was determined by radioallergosorbent assay (RAST). 

### Statistical analysis

Statistical analyses were done in part with JMP8.0 (SAS Institute Inc.). McNemar’s chi square tests were used for paired comparisons. Variables with a univariate P value of <0.2 were included in a logistic regression model. Multinomial logistic regression to analyze a 1-1 matched case-control study was performed using IBM SPSS Statistics Version 19 (2010, SPSS Inc).

## Results

### Clinical manifestations of urticaria

Tables 1 and 2 describe the characteristics of cases and controls. The mean age of the cases and controls was 3.8 years (Table 3) with a range from 1 month to 15 years. Twenty of 37 (54%) cases had a single episode of acute urticaria, and the remaining 17 (46%) had recurrent acute urticaria. While acute urticaria prevailed in children younger than 2 or older than 6 years of age, recurrent urticaria occurred more frequently in children between 2 and 6 years (p <0.05). Urticaria was associated with other clinical manifestations in 27 cases (73%). 

**Table 1:** Clinical manifestations and infections in children with acute or acute recurrent urticaria.

**Cases**	**Gender**	**Age (years)**	**Urticaria**	**Clinical disease concurrent with urticaria**	**Infections concurrent with urticaria**	**Positive diagnostic tests**	**Allergic data**	**Hospital days**
1.	M	1.1	Acute	Herpes labialis	HSV-1	IgM	RAST: cow's milk 0.55 kU/L	none
2.	M	1.3	Acute	Pneumonia	none	None	Atopic dermatitis	none
3.	M	4	Acute	Gastroenteritis	GAS^*^	ASO 1375 IU/ml	Total IgE 181 kU/L	6
					HHV-6	DNA saliva		
					Rotavirus	Antigen in stools		
4.	F	7	Acute recurrent	Pneumonia	*M. pneumoniae*	IgM		8
					EBV	IgM		
5.	M	7.5	Acute recurrent	Pharyngotonsillitis	EBV	DNA blood/saliva	RAST: cow's milk 0.36 kU/L	none
6.	F	11	Acute	Pneumonia	*M. pneumoniae*	IgM		5
7.	F	5.4	Acute recurrent	none	CMV	DNA urine		none
					ParvovirusB19	IgM		
8.	M	6.2	Acute recurrent	Pharyngotonsillitis Abdominal pain	GAS	ASO 690 IU/ml	Atopic dermatitis	none
					H.Pylori	Antigen in stools		
					EBV	IgM		
9.	M	4.8	Acute recurrent	none	none	none		3
10.	M	2	Acute recurrent	Pneumonia	Adenovirus	IgM	Atopic dermatitis	4
11.	F	2	Acute	none	HHV-6	DNA saliva	Rhinitis	none
12.	F	11	Acute	Pharyngotonsillitis	CMV	IgM	Wheezing Prick: *Graminaceae*+	none
13.	M	3.6	Acute	Gastroenteritis	CMV	IgM	none	3
14.	M	1.6	Acute	Nasal herpes	EBV	IgM, DNA blood/saliva	none	5
					HSV-1	IgM		
15.	M	4.5	Acute	Abdominal pain	none	none	Wheezing	3
16.	F	2.5	Acute recurrent	Otitis	HHV-6	DNA blood/saliva	Wheezing	4
17.	M	7	Acute recurrent	Epididymitis	none	none	none	none
18.	F	2.2	Acute recurrent	none	HHV-6	DNA blood/saliva	none	4
					ParvovirusB19	IgM		
19.	F	2	Acute	Pharyngotonsillitis	none	none	none	none
20.	M	2.3	Acute recurrent	none	none	none	none	none
21.	F	0.5	Acute recurrent	none	EBV	DNA saliva	RAST: cow's milk 2.1 kU/L	none
22.	F	8.1	Acute	Pharyngotonsillitis	none	none	Rhinitis Prick: *Graminaceae*+	none
23.	M	3	Acute	none	none	none	none	none
24.	F	2.4	Acute recurrent	Pharyngotonsillitis	CMV	DNA blood/saliva	Atopic dermatitis	none
					HHV-6	IgM		
25.	F	0.4	Acute recurrent	none	HHV-6	DNA saliva	Atopic dermatitis	none
26.	M	0.7	Acute recurrent	Pharyngotonsillitis Gastroenteritis	HHV-6	DNA blood/saliva	none	none
27.	F	1.0	Acute	Wheezing	none	none	none	none
28.	F	3	Acute	Pharyngotonsillitis	HHV-6	IgM	none	none
29.	M	1.0	Acute	Pharyngotonsillitis	none	none	none	none
30.	M	1.3	Acute	none	none	none	none	none
31.	M	8	Acute	none	CMV	IgM	none	none
32.	M	1.3	Acute	Gingivostomatitis	GAS	ASO 601 IU/ml	RAST: *D. pteronissinus* 1.32 kU/L; *D. farinae* 0.8 kU/L	6
					HSV-1	IgM		
33.	F	1	Acute	Urinary tract infection	HHV-6	IgM	none	none
34.	M	2	Acute	none	CMV	IgM, DNA saliva	RAST: egg 0.82 kU/L; cow's milk 0.76 kU/L	none
35.	M	5.8	Acute recurrent	Aphtous stomatitis	GAS	ASO 552 IU/ml	none	none
					HSV-1	IgM		
36.	F	0.3	Acute recurrent	none	CMV	IgM, DNA saliva	RAST: cow's milk 1.63 kU/L	
37.	F	8.1	Acute recurrent	Pharyngotonsillitis	GAS	Pharyngeal swab: GAS, ASO 477 IU/ml	none	5
					HHV-6	DNA saliva		
					CMV	IgM		

GAS = Group A β-hemolytic Streptococcus

**Table 2:** Characteristics of healthy controls.

**Controls**	**Gender**		**Age (years)**	**Subclinical findings**	**Positive infectious tests**	**Allergic data**
1.	M		1.9	none	none	none
2.	F		1.1	none	none	none
3.	M		3.8	none	none	none
4.	M		7.1	GAS	ASO 502	none
5.	F		7.8	none	none	none
6.	M		10.1	GAS	ASO 748	none
7.	M		5.1	none	none	none
8.	M		6	GAS	ASO 630	none
9.	M		4.3	none	none	none
10	F		2.5	GAS	ASO 440	none
				HHV-6	DNA saliva	
11.	F		2.1	CMV	DNA saliva urine	none
12.	F		11	none	none	none
13.	M		3.2	none	none	none
14.	F		1.3	none	none	none
15	M		3.8	none	none	RAST: cow's milk 2.9 kU/L
16.	F		2.8	none	none	none
17	F		7.6	none	none	none
18.	F		2.3	none	none	none
19.	F		2.1	HHV-6	DNA saliva	none
20.	M		2.7	Adenovirus	IgM	none
21.	M		0.7	none	none	Atopic dermatitis
22.	M	9.5		GAS	ASO 361	RAST: cow's milk 1.6 kU/L
				CMV	DNA saliva/urine	
23.	M		2.8	GAS	ASO 383	none
24	F		2.8	none	none	none
25.	M		0.8	none	none	none
26.	M		0.9	none	none	none
27.	M		1.4	none	none	none
28	M		3.1	none	none	Atopic dermatitis, Asthma
29.	M		1.3	none	none	Atopic dermatitis Prick test: *D. pteronissinus*
30.	M		1.7	none	none	none
31.	M		7.4	GAS	ASO 893	none
32.	F		11.6	none	none	none
33.	M		1.9	none	none	none
34.	M		0.1	none	none	none
35.	F		5.7	none	none	none
36.	F		0.6	none	none	none
37.	M		10.4	none	none	Rhinoconjunctivitis RAST: *P. officinalis* 3.45 kU/L

**Table 3:** Comparison of cases and controls for possible risk factors for urticaria.

Characteristics	Cases Disease N= 37	Controls No disease N =37	Univariate P value	Multivariate P value (adjusted odd ratio, 95% confidence interval)
No of male cases	20	23	0.63	Not done
Mean age + SD of case and controls (years) -matching criteria	3.7+3.0	3.9+3.0	Not done	Not done
No. of children with CMV	8	2	0.12	0.10 (3.5, 0.67-19)
No. of children with HSV	4	0	0.13	0.019*
No. of children with EBV	5	0	0.07	0.017*
No. of children with HHV-6	10	2	0.043	0.019 (5.1, 1.06-24.3)
No. of children with Adenovirus	1	1	0.48	Not done
No .of children with any herpes infection (CMV, HSV, EBV, HHV)	24	4	0.0003	Not done
No. of children with Parvovirus	2	0	0.48	Not done
No. of children with *M. Pneumoniae*	2	0	0.48	Not done
No. of children with Rotavirus	1	0	1.00	Not done
Number of children with group A Streptococcal infections	5	7	0.75	Not done
No. of children with RAST/Prick	8	4	0.68	Not done
No. of children with allergy or atopy	5	3	1.00	Not done
No. of children with wheezing or rhinitis	5	1	0.22	Not done

^*^ Too few subjects were infected to calculate an accurate odds ratio***.***

### Laboratory and immunologic findings

Fifteen cases had elevated leukocytes (>9.1×10^3^/mm^3^) which ranged from 4.47×10^3^/mm^3^ to 34.7×10^3^/mm^3^ (mean values 10.27×10^3^/mm^3^) and of these 14 also had lymphocytosis. Ten cases had eosinophilia (> 1500 eosinophils/mm^3^). Seven cases had increased platelets (> 450×10^3^/mm^3^), 7 had an elevated ESR; 2 had an elevated CRP (>0.5 mg/l); 3 had abnormal ALT and AST levels . Immunoglobulin, C3 and C4 levels were normal in all cases. 

### Risk factors

Ten cases (37%) had past allergic manifestations (Table 1). Six cases had increased levels of specific IgE antibodies towards cow’s milk, egg, *Dermatophagoides pteronissimus/farinae*. One case had an increased level of total IgE antibodies. Only two cases had positive skin prick tests to *Graminaceae*. Ibuprofen was the possible cause of acute urticaria in only one case.

### Virologic findings

Specific infections were diagnosed in 26 of 37 cases and among 9 of 37 control children (70% vs 24%; P=0.0002). Of infected cases, 25 had viral infections, six had viral-bacterial co-infections, six were infected with two viruses, and one had *M. pneumoniae* infection. Two or more infections occurred in 7 of 17 cases (41%) who had recurrent urticaria compared with two or more infections in 2 of 20 cases (10%) who had only one episode of urticaria (P =0.05). 

The cases, compared to the age matched controls, had a higher prevalence of herpesvirus infections: single or concomitant herpesvirus infections occurred in 24 cases and in 4 controls (65% vs 11 %, p=0.0003, Table 3). Cases had 27 herpesvirus infections, including 10 HHV-6 infections, 8 CMV infections, 5 EBV infections, and 4 HSV-1 infections (Table 1). In 15 cases, 16 primary herpesvirus infections were identified by high levels of specific IgM and increasing IgG antibodies. One case was infected with two primary herpesvirus infections. In 9 cases, 11 active nonprimary herpesvirus infections were diagnosed by concomitant detection of specific DNA and high levels of IgG. Two cases had concomitant primary and nonprimary herpesvirus infections. 

The only infections significantly associated with urticaria were infections with one of the four herpesviruses, CMV, EBV, HSV, or HHV-6 (Table 3). In a multinomial regression analysis infections by HHV-6, HSV, and EBV were each independently predictive of acute or recurrent urticaria and CMV approached statistical significance (P = 0.1). None of the other infections whether viral or bacterial approached statistical significance (Table 3). Among 24 cases with a herpesvirus infection, 13 (54%) had recurrent acute urticaria compared with recurrent acute urticaria in 4 of 13 (31%) cases without a herpesvirus infection (p = 0.3). Atopy or allergies were not statistically (p =0.3) more frequent among cases with herpesvirus infections (13/17 = 76%) than cases without (11/20 =55%). 

## Discussion

Acute urticaria occurs in 15% to 25% of people at a some point in their life, being associated with transudation of fluid from skin blood vessels and release of mediators from cutaneous mast cells and basophils [14] The release of mast cell-derived mediators may be caused by both immune and non immune mechanisms [15]. The association of acute urticaria with atopy is common, being found up to 50% of the cases and occasionally associated with an increase in total IgE level, which may represent a nonspecific marker of the immune mechanisms involved in urticaria rather than a sign of underlying atopy [12]. 

In children, acute urticaria is often considered as a food or drug allergy, leading to frequently unnecessary diet restrictions or elimination of drugs and administration of antihistaminic or corticosteroids. Identification of the true casual factors for urticaria would be very helpful in the management and prognosis. Possible causes, however, are numerous having been reported in between 21% to 83% of cases [2,16]. This high variability is due to the various criteria used in evaluating the causes and the enrollment criteria. The various criteria used have included acute or chronic infections, non allergic hypersensitivity reactions to foods and drugs, and autoimmunity mediated by functional autoantibodies directed against the IgE receptors [13,17–19]. Between 37% and 58%, of children with acute urticaria also have infections, usually virus-induced upper respiratory infections [2,3]. Viral infections have also been associated to the onset of atopic manifestations and an increase in systemic IgE levels [20]. Infections, however, frequently also occur in patients with drug allergies making it difficult to distinguish between the two possible causal factors [16].

Ours is the first prospective age-matched case-control study to evaluate the association between acute or recurrent acute urticaria and a broad range of infectious causes in children. One previous study reported a 32% rate of *M. pneumoniae* infections among 65 children but a so called control group of 49 children with respiratory infections was not tested for *M. pneumoniae*. We found no evidence that acute urticaria was associated with Mycoplasma or streptococcal or respiratory viral infections. We did however observed that the onset of acute urticaria was strongly associated with an herpesvirus infection, including children who developed recurrent acute urticaria. Herpesviruses can persist long life alternating latent and reactivation phases, thus primary or reactivated herpesvirus infections may be associated with acute or recurrent acute urticaria. Cellular immunity controls herpesvirus infections and both primary and recurrent infections are associated with altered cell-mediated responsiveness and production of a number of cytokines [21]. As occurs in virus-induced asthma exacerbations after viral induction of mast cell activation, the activation of mast cells may be involved in herpesvirus-induced diseases, including the urticaria in genetically predisposed children [15,22]. 

HHV-6, CMV and EBV infections are common in children. In particular the seroprevalence rate of HHV-6 is between 80% and 100% by the age of 3 years [23]. HHV-6 usually causes exanthema subitum but can affect the immunosurveillance system because of its tropism for T lymphocytes, monocytes/macrophages, and dendritic cells. In fact, HHV-6 stimulates the production of interferon-α and tumor necrosis factor-α, and upregulates NK cell cytotoxicity via interleukin-15 [24,25]. CMV infection is common both pre and postnatally with an overall prevalence of between 15% and 25% among children, and also has many effects on the immune system mostly via cell-mediated immunity [21]. Eight children had persistent CMV DNA detection for more than 3 months after acute urticaria: six of these children developed recurrent acute urticaria. EBV and HSV-1 infections also stimulate cellular and antibody immune responses [21,26]. Caubet et al. recently reported on a viral trigger, including acute EBV infections, for the urticaria or a red rash in 54/82 (66%) patients presenting to an emergency room with a negative oral challenge test (OCT). Interestingly, 3 of the patients with a positive OCT (50%) had findings suggestive of an acute or recent EBV infection [27]. 

Herpesviruses are a component of the human "microbiome" since they are adapted to lifelong infection of their hosts. Recent evidence suggest a possible role for herpesviruses in allergy and atopy [9]. Our results with urticaria and those with allergic and atopic reactions suggest physicians caring for allergic and atopic populations should be familiar with the manifestations of herpes-related disease [27,28].

Because herpesvirus infections are common among children, possible limitations of our study include the small sample size and the practical limitation of only being able to obtain, in most cases, single specimens for diagnostic testing. Perhaps more subjects and repetitive testing would skew the results. Nevertheless with a significantly higher rate than controls, we observed a herpesvirus infection in 65% of our patients which suggests herpesviruses may be component of the process involved in development of acute or recurrent acute urticaria. 

## References

[1] Wedi B, Kapp A (2008). Urticaria and angioedema. In: Mahmoudi M Allergy: Practical Diagnosis and Management. New York: McGraw Hill p. 84-94.

[2] Sackesen C, Sekerel BE, Orhan F, Kocabas CN, Tuncer A et al. (2004) The etiology of different forms of urticaria in childhood. Pediatr Dermatol 21: 102-108. doi:10.1111/j.0736-8046.2004.21202.x. PubMed: 15078346. 15078346

[3] Kanokvalai, Yodmanee C, Sukhum J (2008) Acute urticaria: etiologies, clinical course and quality of life. Asian Pac J Allergy Immunol 26: 1-9. PubMed: 18595524. 18595524

[4] Wu C-C, Kuo H-C, Yu H-R, Wang L, Yang KD (2009) Association of acute urticaria with *Mycoplasma* * pneumoniae* infection in hospitalized children. Ann Allergy Asthma Immunol 103: 134-139. doi:10.1016/S1081-1206(10)60166-4. PubMed: 19739426. 19739426

[5] Cherry JD, Lerner AM, Klein JO, Finland M (1963) Coxsackie A9 infections with exanthemas, with particular reference to urticaria. Pediatrics 31: 819-823. PubMed: 14020634. 14020634

[6] Dienstag JL, Rhodes AR, Bhan AK, Dvorak AM, Mihm MC Jr et al. (1978) Urticaria associated with acute viral hepatitis type B: studies of pathogenesis. Ann Intern Med 89: 34-40. doi:10.7326/0003-4819-89-1-34. PubMed: 352219. 352219

[7] Frick OL, German DF, Mills J (1979) Development of allergy in children: I. Association with virus infections. J Allergy Clin Immunol 63: 228-241. doi:10.1016/0091-6749(79)90106-4. PubMed: 85648. 85648 PMC7133211

[8] Schuller DE, Elvey SM (1980) Acute urticaria associated with streptococcal infection. Pediatrics 65: 592-596. PubMed: 6987606. 6987606

[9] Scully LJ, Ryan AE (1993) Urticaria and acute hepatitis A virus infection. Am J Gastroenterol 88: 277-278. PubMed: 8380951. 8380951

[10] Lipsker D, Boeckler P (2006) Acute urticaria and dry cough with interstitial pneumonia: a clue for the diagnosis of primary parvovirus B19 infection. Clin Exp Dermatol 31: 473-474. doi:10.1111/j.1365-2230.2006.02087.x. PubMed: 16681615. 16681615

[11] McMahon AW, Iskander JK, Haber P, Braun MM, Ball R (2008) Inactivated influenza vaccine (IIV) in children <2 years of age: examination of selected adverse events reported to the Vaccine Adverse Event Reporting System (VAERS) after thimerosal-free or thimerosal-containing vaccine. Vaccine 26: 427-429. doi:10.1016/j.vaccine.2007.10.071. PubMed: 18093701. 18093701

[12] Zuberbier T (2003) Urticaria. Allergy 58: 1224-1234. doi:10.1046/j.1398-9995.2003.00327.x. PubMed: 14616095. 14616095

[13] Liu YR, Yang KC, Chou CC, Chang YJ, Wu HP (2008) First attack of acute urticaria in pediatric emergency department. Pediatr Neonatol 49: 58-64. doi:10.1016/S1875-9572(08)60014-5. PubMed: 18947000. 18947000

[14] Kanwar AJ, Greaves MW (1996) Approach to the patient with chronic urticaria. Hosp Pract 31: 175-189. PubMed: 8596002. 8596002

[15] Corne JM, Holgate ST (1997) Mechanism of virus induced exacerbations of asthma. Thorax 52: 380-389. doi:10.1136/thx.52.4.380. PubMed: 9196525. 9196525 PMC1758527

[16] Mortureux P, Léauté-Labrèze C, Legrain-Lifermann V, Lamireau T, Sarlangue J et al. (1998) Acute urticaria in infancy and early childhood: a prospective study. Arch Dermatol 134: 319-323. doi:10.1001/archderm.134.3.319. PubMed: 9521030. 9521030

[17] Wedi B, Raap U, Kapp A (2004) Chronic urticaria and infections. Curr Opin Allergy Clin Immunol 4: 387-396. doi:10.1097/00130832-200410000-00010. PubMed: 15349038. 15349038

[18] Zuberbier T, Chantraine-Hess S, Hartmann K, Czarnetzki BM (1995) Pseudoallergen-free diet in the treatment of chronic urticaria - a prospective study. Acta Derm Venereol 75: 484-487. PubMed: 8651031. 8651031 10.2340/0001555575484487

[19] Hide M, Francis DM, Grattan CEH, Hakimi J, Kochan JP et al. (1993) Autoantibodies against the high-affinity IgE receptor as a cause of histamine release in chronic urticaria. N Engl J Med 328: 1599-1604. doi:10.1056/NEJM199306033282204. PubMed: 7683772. 7683772

[20] Kumar A, Grayson MH (2009) The role of viruses in the development and exacerbation of atopic disease. Ann Allergy Asthma Immunol 103: 181-186. doi:10.1016/S1081-1206(10)60178-0. PubMed: 19788013. 19788013 PMC7129158

[21] Smith C, Khanna R (2013) Immune regulation of human herpesviruses and its implications for human transplantation. Am J Transplant Suppl 3: 9-23. PubMed: 23347211. 10.1111/ajt.12005 23347211

[22] McAlpine SM, Enoksson M, Lunderius-Andersson C, Nilsson G (2011) The effect of bacterial, viral and fungal infection on mast cell reactivity in the allergic setting. J Innate Immun 3: 120-130. doi:10.1159/000323350. PubMed: 21242671. 21242671

[23] Levy JA, Ferro F, Greenspan D, Lennette ET (1990) Frequent isolation of HHV-6 from saliva and high seroprevalence of the virus in the population. Lancet 335: 1047-1050. doi:10.1016/0140-6736(90)92628-U. PubMed: 1970369. 1970369

[24] Ablashi DV, Salahuddin SZ, Josephs SF, Imam F, Lusso P et al. (1987) HBLV (or HHV-6) in human cell lines. Nature 329: 207-211. doi:10.1038/329207a0. PubMed: 3627265. 3627265

[25] Arena A, Capozza AB, Di Luca D (1996) Role of IFN gamma on TNF alpha, IL-1 beta and IL-6 release during HHV-6 infection. New Microbiol 19: 183-191. PubMed: 8841033. 8841033

[26] Nilsson C, Larsson Sigfrinius AK, Montgomery SM, Sverremark-Ekström E, Linde A (2009) Epstein-Barr virus and cytomegalovirus are differentially associated with numbers of cytokine-producing cells and early atopy. Clin Exp Allergy 39: 509-517. doi:10.1111/j.1365-2222.2008.03147.x. PubMed: 19055650. 19055650

[27] Caubet JC, Kaiser L, Lemaître B, Fellay B, Gervaix A et al. (2011) The role of penicillin in benign skin rashes in childhood: a prospective study based on drug rechallenge. J Allergy Clin Immunol 27: 218-222. 10.1016/j.jaci.2010.08.025 PMC7126001 21035175

[28] Dreyfus DH (2013) Herpes viruses and the microbiome. J Allergy Clin Immunol 132: 1278-1286. doi:10.1016/j.jaci.2013.02.039. PubMed: 23611298. 23611298

